# Pre-concentration of microRNAs by LNA-modified magnetic beads for enhancement of electrochemical detection

**DOI:** 10.1038/s41598-021-99145-8

**Published:** 2021-10-04

**Authors:** Serife Ustuner, Mark A. Lindsay, Pedro Estrela

**Affiliations:** 1 Department of Electronic and Electrical Engineering, University of Bath, Bath, BA2 7AY UK; 2 Centre for Biosensors, Bioelectronics and Biodevices (C3Bio), University of Bath, Bath, BA2 7AY UK; 3 Centre for Sustainable and Circular Technologies, University of Bath, Bath, BA2 7AY UK; 4 Department of Pharmacy and Pharmacology, University of Bath, Bath, BA2 7AY UK

**Keywords:** Isolation, separation and purification, Sensors and probes, Biomedical engineering, Electrochemistry

## Abstract

MicroRNAs are extremely promising candidates for early cancer diagnosis and prognosis. The levels of circulating microRNAs provide valuable information about cancer disease at its early stages. However, the levels of microRNAs that need to be detected are extremely low and difficult to discriminate from a large pool of oligonucleotides. There is the need for accurate, rapid and sensitive detection methodologies for detection of microRNAs. We developed electrochemical impedance spectroscopy peptide nucleic acid (PNA)-based sensors that can detect miRNAs in diluted serum with a limit of detection of 0.38 fM. In order to further improve the accuracy and reliability of the sensors, we developed an assay using magnetic beads for simple and rapid fishing of target microRNAs from solution and its pre-concentration prior to electrochemical detection. Our methodology utilizes magnetic beads for the capture of the target microRNA from solution and brings the concentrated sample to the sensor surface. We modify the magnetic beads with locked nucleic acids (LNA), which have high affinity and specificity to their complementary microRNA sequence. The separated and concentrated microRNA is then detected using the PNA-based sensors. By exposing the sensing electrodes only to the captured microRNAs, interferences from other nucleotides or biomolecules from the sample are eliminated.

## Introduction

MicroRNAs (miRNAs) are a class of 19–24 nucleotides, noncoding RNAs, which regulate the expression of nearly one third of all human genes^1–3^. Within the context of cancer disease development and progression, epigenetic alterations are considered as one of the key drivers. Epigenetic alterations are shown to take place far more frequently than genetic mutations and often appear in early stages of tumorigenesis^4,5^. The term epigenetic here refers to all heritable changes in gene expression but eliminates the alterations in a DNA sequence. To date, miRNAs remain the most epigenetic alteration in circulation, which is a demanded property for a diagnostic and prognostic biomarker. Moreover, miRNAs are shown to be stable in blood due to their packaging into exosomes that protect them from degradation, while other circulating nucleic acids, such as DNA, are cleared from circulation rapidly.

MiRNAs have been a particular area of interest for oncology research since their discovery and following the evidence suggesting that they are involved in the pathogenesis of cancer, in most cases by controlling the translation of oncogenes and tumor suppressors^6–8^. The levels of miRNAs in circulating blood were discovered to show fluctuations in different cancer states^9^, making them potential minimally-invasive diagnostic markers for cancer.

In pancreatic cancer, a number of miRNAs have been found to be either up-regulated or down-regulated^10,11^. In particular, miR-21 was found to be upregulated in pancreatic cancer patients^12,13^. A systematic review and meta-analysis evaluated the prognostic role of miR-21 in patients with pancreatic cancer and found that upregulation is associated with poorer overall, disease-free, and progression-free survival as well as with a higher rate of metastatic lymphnodes and poor differentiated tumors^14^.

Prior to the implementation of miRNA sensing technology for clinical applications, there are number of technical challenges to be addressed. The most critical one is the low and undefined levels of miRNAs in blood to detect^15^. Changes to specific miRNA levels in serum/plasma have been defined as up- or down- regulation between healthy controls and patients with disease state but with little literature stating the actual concentration of miRNA in healthy biological samples and the change of this concentration in disease state. This could be attributed to the lack of standardized protocols for separation of miRNAs, their processing and quantification. Hence, this makes it extremely difficult to carry out quantitative comparisons between the studies reported in literature due to the slightly different conditions of each study. Nonetheless, there are suggestions that miRNAs in blood might need to be detected in the pM to fM ranges. Other challenges are the short sequence length of miRNAs and the high sequence homology within the family of miRNAs. Hence, there is the requirement for highly specific sensors that can distinguish between miRNAs that possess a few bases mismatch.

Magnetic beads hold many applications and are a well-known solid support for electrochemical detection^16–18^ and efficient nucleic acid separation^19^. The adoption of magnetic beads for the electrochemical detection of miRNAs has been exploited in different studies. Bettazzi et al*.*^20^ obtained a detection limit of 7 pM for miR-222 using a multi-step approach: functionalization of streptavidin coated magnetic beads with a biotinylated DNA probe that was specific to the target miRNA; extraction of the target miRNAs from the cell samples and their labelling with biotin; hybridization of biotinylated target with the DNA capture probe on the magnetic bead; labelling of resulting biotinylated hybrid with a streptavidin–alkaline phosphatase conjugate; entrapment on to a screen-printed electrode and measurement of the enzymatic product with differential pulse voltammetry. A similar study reported detection limits as low as femtomolar levels for voltammetric detection of miRNA by designing a magnetic beads-based assay on a multi-channel screen-printed array of electrodes^21^. Several other multi-step studies achieved the creation of highly sensitive miRNA detection platforms^22–26^. Some of these studies are summarized on Table 1. Different amplification strategies for electrochemical and photoelectrochemical sensing of miRNAs have been proposed in the literature^27^, mostly based on the amplification of the signal itself rather than on pre-concentration methods.

**Table 1:** Examples of magnetic beads-based electrochemical detection methodologies for miRNA detection.

Assay description	LOD	Pre-labelling of target	Number of assay steps	Type of capture probe	Reference
Electrochemical detection of miRNA-222 by use of a magnetic bead-based bioassay	7 pM	Yes	7	DNA	^20^
Magnetic bead-based hybridization assay for electrochemical detection of microRNA	fM level	Yes	6	DNA	^23^
Electrically magnetic-controllable electrochemical biosensor for the specific detection of oral cancer-related microRNA	0.22 aM	No	7	RNA	^24^
Magnetobiosensors based on viral protein p19 for microRNA determination in cancer cells and tissues	0.04 nM	No	6	RNA	^25^
Magnetic beads-based sensor with tailored sensitivity for rapid and single-step amperometric determination of miRNAs	10 aM	No	6	DNA	^26^

**Figure 1:**
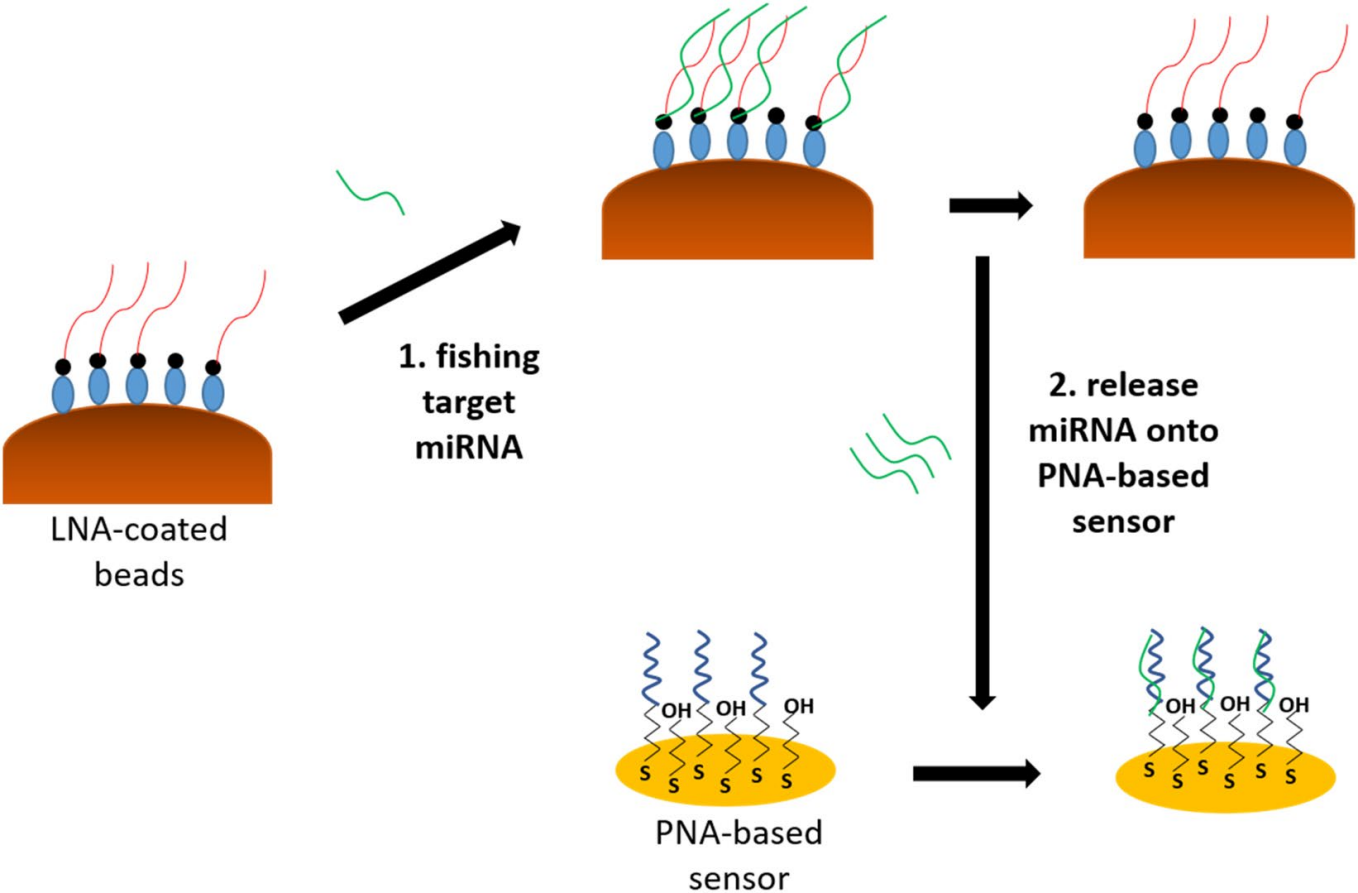
Schematic diagram of the assay: (1) LNA-coated magnetic beads capture target miRNAs from sample; (2) release of the capture miRNA from magnetic beads which are re-hybridized with PNA-probe for electrochemical detection*.*

In order to improve the current methodologies in literature, we designed a new assay that combines the high affinity and separation properties of Locked Nucleic Acid (LNA) modified magnetic beads with the high stability of Peptide Nucleic Acid (PNA) probes on gold electrode surfaces in order to develop a magnetic-bead based direct electrochemical detection of miRNAs from solution. Our methodology initially replaces the DNA/RNA capture probe that is adopted in the literature with a probe that has a higher affinity to miRNAs. LNA is known for displaying extraordinary hybridization affinity towards RNA. The enhanced affinity is a result of the modified chemical structure of LNA^28^ that leads to the high melting points of LNA-RNA^29^ and makes LNA well suited for miRNA detection and analysis for cancer diagnosis. Hence, as schematically illustrated in Fig. 1, a biotinylated LNA probe was adopted and LNA-modified streptavidin coated magnetic beads were utilized for capturing miRNA from solution. After separation of the target miRNA from solution, a number of thermal and chemical denaturation methods were investigated for the most efficient release of the captured miRNA target from the magnetic beads. A PNA-immobilized gold electrode was adopted for the identification of the captured miRNA. PNA oligonucleotides when immobilized on gold electrode surfaces provide exceptional stability and great detection limits for the identification of low concentrations of miRNA in a solution^30,31^. Hence, such a methodology does not only eliminate the pre-labelling step of target but also benefits from the high affinity properties of LNA-probes for capturing low concentrations of miRNA from solution, and their quantification by highly stable PNA-probes. In essence, our approach relies on the following steps: capture of miRNAs by LNA-modified magnetic beads; move beads to sensor area; release miRNA and detect it with PNA-probe electrochemical sensor.

## Materials and methods

### Oligonucleotides

Synthetic biotinylated LNA capture probe was purchased from Eurogentec, UK, in lyophilized form, while HPLC purified synthetic RNA sequences were purchased from Sigma-Aldrich, UK, in lyophilized from. The PNA probe was purchased from Cambridge Research Biochemical, UK. The sequences used are shown on Table 2.

**Table 2:** The miRNA, LNA and PNA sequences used in this study.

miR-21-5p sequence	3′-AGU UGU AGU CAG ACU AUU CGA U-5′
Non-specific miRNA sequence	3′-AGU UGU ACC AGA UAU CCG UAA A-5′
LNA probe	5′-biotin-TEG- T(lnC)A-A(lnC)A-TCA-G(lnT)C-TGA-T(lnA)A-G(lnC)T-(lnA)-3′
PNA probe	SH-C6-AEEA-TTT TCA ACA TCA GTC TGA TAA GCT A

The sequence on the first row corresponds to the miR-21-5p sequence specific to pancreatic cancer. TEG is a 15-atom long linker (tetra-ethyleneglycol), while AEEA is a 9-atom glycol linker (8-amino-3,6-dioxaoctanoic acid). LNA modification sites in the sequence are represented by (ln).

### Preparation of magnetic beads

All chemicals were purchased from Merck Sigma-Aldrich UK unless otherwise stated. Prior to the assay, Pierce™ Streptavidin Magnetic Beads of 1 µm nominal diameter (Thermo Scientific, UK) were washed with 25 mM Tris-buffered saline containing 0.1% Tween-20 (Thermo Scientific, UK). This was carried out by adding 30 µL (0.3 mg) of magnetic beads into a 1.5 mL microcentrifuge tube that was placed on a magnetic stand to attract the beads to the side of the tube. The supernatant was removed with a pipette and discarded. Then 1 mL of wash buffer (Tris-buffered saline) was added to the tube. The tube was inverted several times and a gentle vortex was applied for an even mix. The beads were then collected with a magnetic stand to the side of the tube and the supernatant was removed. The beads were stored in wash buffer until the incubation with the LNA probe was carried out.

This was then followed by washing the beads within the immobilization buffer (10 mM PB buffer, pH 7.3). The tube was inverted several times, and a gentle vortex was applied for an even mix and the beads were collected with the magnetic stand. The supernatant was removed, and the beads were incubated with 300 µL of 1 µM biotinylated LNA-probe solution. 1 µM of biotinylated LNA-probe solution was prepared in 10 mM PB buffer (pH 7.3) prior to immobilization with magnetic beads. The incubation with LNA-probe solution was carried out at room temperature for 1 h with continuous mixing, after which the modified beads were collected by placing the tube on the magnetic stand again and removing the supernatant for further analysis. Then the modified beads were washed with 10 mM PB buffer twice and incubated with 100 µM biotin (prepared in 10 mM PB buffer, pH 7.3) in order to block any remaining streptavidin active sites on the probe-functionalized surface. After biotin blocking, the beads were collected with the magnetic stand, and supernatant was removed for further analysis. This was followed by a final wash of the beads in 10 mM PB buffer.

### Hybridization of LNA-functionalized magnetic beads with target miRNA

300 µL of 1 µM target miRNA was prepared in 10 mM PB (pH 7.3) and heated at 95 °C in a water bath for 5 min prior to incubation. The heating was carried out in order to prevent the formation of hairpins or secondary structures, as the evidence suggests that miRNAs have a tendency to form both hairpin and homoduplex structures in solution^32^. This was followed by incubating the dry LNA-functionalized magnetic beads with the target miRNA solution for 30 min at room temperature, with continuous mixing for the hybridization to take place. In the end, the beads were collected with the magnetic stand, and the remaining solution was removed for further analysis. The beads that contained the LNA–miRNA hybrids were washed twice in 10 mM PB buffer and then a number of thermal/chemical denaturation methods were studied for the most efficient release of the target miRNA from the magnetic beads surface (see also Supplementary Information). Following the denaturation step, the beads were separated from the solution by use of the magnetic stand and the solution that contained the captured miRNA target was removed for electrochemical detection on PNA-probes.

### LNA–miRNA denaturation

Thermal denaturation (release) of the miRNAs from the LNA-modified magnetic beads was performed by heating 300 µL of solution in a water bath at 95 °C for 10 min.

Chemical denaturation of the LNA–miRNA duplex was performed with 1 M NaOH. The stock solution of NaOH (10 M) was prepared upon dissolving 4 g of NaOH (Sigma-Aldrich) in 10 mL of Milli-Q water. The NaOH was diluted to 1 M in Milli-Q for the assays. 300 µL of 1 M NaOH were added to the tube containing LNA-functionalized magnetic beads previously hybridized with target miRNA. The mixture was incubated at ambient temperature for 10 min prior to measurements with UV spectrophotometry.

### UV-Spectrophotometer measurements

A Shimadzu UV-1800 with Thermal Melt Analysis System was adopted in order to validate the assay steps. The solutions of LNA-probe and target miRNA prior to their bio-functionalization and hybridization on magnetic beads surface were analyzed by UV-spectrophotometry. This was followed by the analysis of the final solution of target miRNA after its capture on the magnetic beads surface and release. 8-series micro cells with silicone plugs that fit inside the spectrophotometer were adopted in order to measure the samples; this has the benefit of allowing the user to analyze 8 different samples during one measurement. The amount of light that is absorbed by the solution is dependent on the concentration, the path length of the light through the cuvette and how well the analyte absorbs the light at a certain wavelength. It is known that single or double stranded DNA, RNA or LNA oligonucleotides, absorb ultraviolet (UV) light due to the existence of heterocyclic rings of the nucleotides. The absorbance levels are usually observed to be around 260 nm^33,34^ and these absorbance properties can be adopted for their quantification^35^. Hence, UV spectrophotometry was run within the region of 190–800 nm, which allows enough room to identify the LNA-probe, initial target miRNA, and the captured target miRNA. Initially, a baseline measurement was carried out by adding 80 µL of buffer solution (10 mM PB) to each of the micro-cells which sets a reference baseline prior to the actual measurements of nucleic acids within the same buffer. Following the baseline measurement, each micro-cell was emptied and re-filled with 80 µL of solutions which contained the nucleic acids prepared in the same buffer (10 mM PB) for the analysis.

### Electrochemical detection of captured miRNA

Gold working electrodes (CH Instruments, USA) were mechanically polished for 2 min with 1 µM diamond solution (Buehler, USA) on a polishing pad (BASi, USA). This was followed by mechanical polishing using alumina slurry with decreasing particle sizes (starting with 1 µm, then 0.3 µm, and finishing with 0.05 µm). Polishing was carried out for at least 2 min in each particle size. In between each polishing step, 5 min of sonication in ethanol and rinsing in Milli-Q water were performed to remove the residues. After the final polishing, 10 min of sonication in ethanol followed by 10 min in Milli-Q water was carried out. Electrodes were then rinsed with Milli-Q water and electrochemically cleaned in 0.5 M H_2_SO_4_ (Fisher Scientific, UK) by scanning the potential between the oxidation and reduction of gold, 0 V and + 1.5 V vs. an Ag/AgCl reference electrode (BASi, USA), for 50 cycles until the voltammogram graphs suggest no further changes. Finally, the electrodes were rinsed with Milli-Q water and dry cleaned under a nitrogen gun.

Clean electrodes were then co-immobilized with the thiolated ssPNA probe sequence and 6-mercapto-1-hexanol (MCH) in 50% dimethyl sulfoxide (DMSO) 50% ultra-pure water (v/v) immobilization solution for 16 h in a humidity chamber. After immobilization, electrodes were rinsed with excess Milli-Q water to remove any unattached thiols and were backfilled with 1 mM MCH for one hour in order to ensure complete thiol coverage. Electrodes were then rinsed with excess Milli-Q water and placed in the measurement buffer for 2 h to stabilize the SAM.

The experiments were carried out with a PalmSens4 compact potentiostat (PalmSens, Netherlands) and a three-electrode cell system: Ag/AgCl (KCl) reference electrode connected via a salt bridge filled with 10 mM phosphate buffer (pH 7.3), Pt counter electrode (ALS, Japan), and gold working electrode (2.0 mm diameter). The Faradaic EIS was conducted in 10 mM PB (pH 7.3) measurement buffer containing 10 mM of the ferro/ferricyanide ([Fe(CN)_6_])^3−/4−^ redox couple over a frequency range from 100 kHz to 100 mHz, with a 10 mV ac voltage superimposed on a dc bias voltage of 0.2 V vs. Ag/AgCl (corresponding to the formal potential of the redox couple).

## Results and discussion

### Electrochemical impedance spectroscopy for detection of microRNA in serum

Electrochemical Impedance Spectroscopy (EIS) in Faradaic mode was adopted to investigate the optimized mole fraction of PNA to total thiol (thiolated PNA plus MCH) in the immobilization solution for enhancement of the hybridization signal. Thiol-modified PNA probe and mercaptohexanol were co-immobilized onto gold electrodes and the charge transfer resistance for the electrode surface was determined upon interaction with 10 nM and 100 nM of miR-21-5p in 10 mM PB (pH 7.3). The best hybridization efficiencies were obtained upon adopting 1:10 and 1:15 ratios of PNA to MCH on the surface (see Supplementary Information). When the probe density was reduced further to the 1:20 ratio, the hybridization efficiency drops due to insufficient amount of probe on the electrode surface^36^. Although 1:15 ratio performed the highest hybridization efficiency, it has performed to be a less stable probe upon incubation with blank compared to the 1:10 ratio. Hence, a 1:10 ratio was adopted for further assays of miR-21-5-p detection.

**Figure 2:**
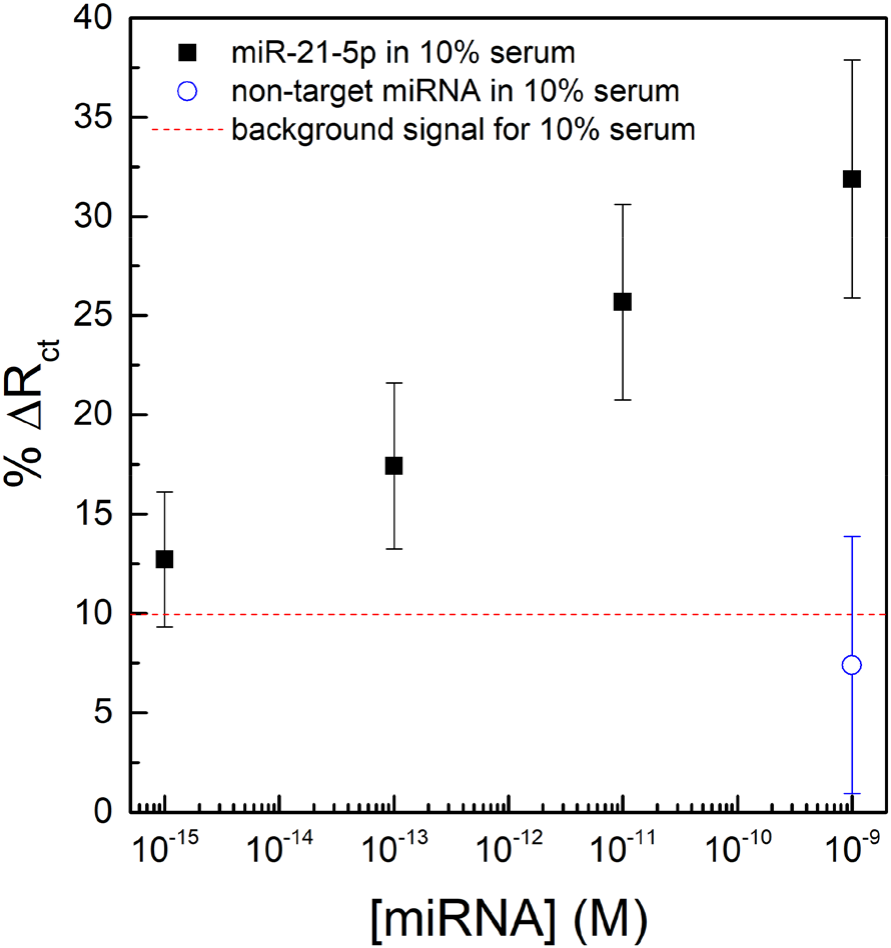
*R*_ct_ variations upon incubations with 10% serum (red line), miR-21-5p diluted in 10% serum (black squares) and with 1 nM of a non-specific miRNA in 10% serum (open blue circle). The error bars represent the standard deviation for three separate electrodes.

The optimized sensor with 1:10 ratio of PNA to MCH on gold electrode surface was then challenged with different concentrations of miR-21-5p spiked in 10% serum (diluted in 10 mM PB), as well as with a high concentration of non-specific miRNA (1 nM) to check for specificity. Figure 2 displays the changes in *R*_ct_ upon incubation of the PNA-based sensor with 10% serum, miR-21-5p in 10% serum in concentrations between 1 fM and 1 nM, and 1 nM of non-specific miRNA in 10% serum. A very low limit of detection was calculated as 0.38 fM (using value for blank + 3 × standard deviation of blank^37^). Although some signal is obtained with just the serum, a clear signal differentiation is observed even for concentrations as low as 1 fM of miR-21-5p in 10% serum.

### LNA-modified magnetic beads for fishing and pre-concentration of microRNA

In order to further improve the sensitivity of detection in complex solutions such as serum, there is the need for additional assay steps for amplification of the detection signal or further improvement of surface chemistry for elimination of the non-specific interactions on the sensor surface. Herein, we develop a new methodology for the feasibility of simple separation and pre-concentration of miRNA from a sample prior to its direct electrochemical detection on a PNA-immobilized gold electrode surface (Fig. 1).

#### UV-Spectrophotometry for validation of design steps

UV–Vis spectroscopy was initially adopted for the identification of the solutions in order to validate the feasibility of each design step. UV measurements provided the absorbance levels for the solutions containing the probe and the target (LNA and miRNA) adopted in the experiments, and the residue solutions of magnetic beads before/after their functionalization with the LNA-probe, hybridization with the target sequence and upon thermal or chemical denaturation. An absorbance peak at around 260 nm is expected for nucleic acids^33^. Hence, a similar absorbance peak for the final solution of target miRNA captured by the magnetic beads would prove the success of the methodology for fishing for miRNA from solution.

Prior to analysis, we tested the thermal stability of the streptavidin magnetic beads modified with biotinylated LNA, under extreme heat conditions (95 °C for 10 min). Such a study is crucial in order to make sure that the stability of streptavidin–biotin binding (between the beads and the LNA-probe) is conserved under the high temperatures normally used for oligonucleotide dehybridization that could be used for the release of the miRNAs from the LNA-modified magnetic beads. An inability of the magnetic beads-LNA binding to resist high temperatures could result in the release of the LNA probe together with the target miRNA from the magnetic beads’ surface, hence leading to false signals in the final solution for captured target miRNA. Although the biotin, after binding streptavidin, increases the thermal stability of streptavidin significantly^38^, there are studies that reported the reversible breakage of the bond under elevated temperatures^39^.

**Figure 3:**
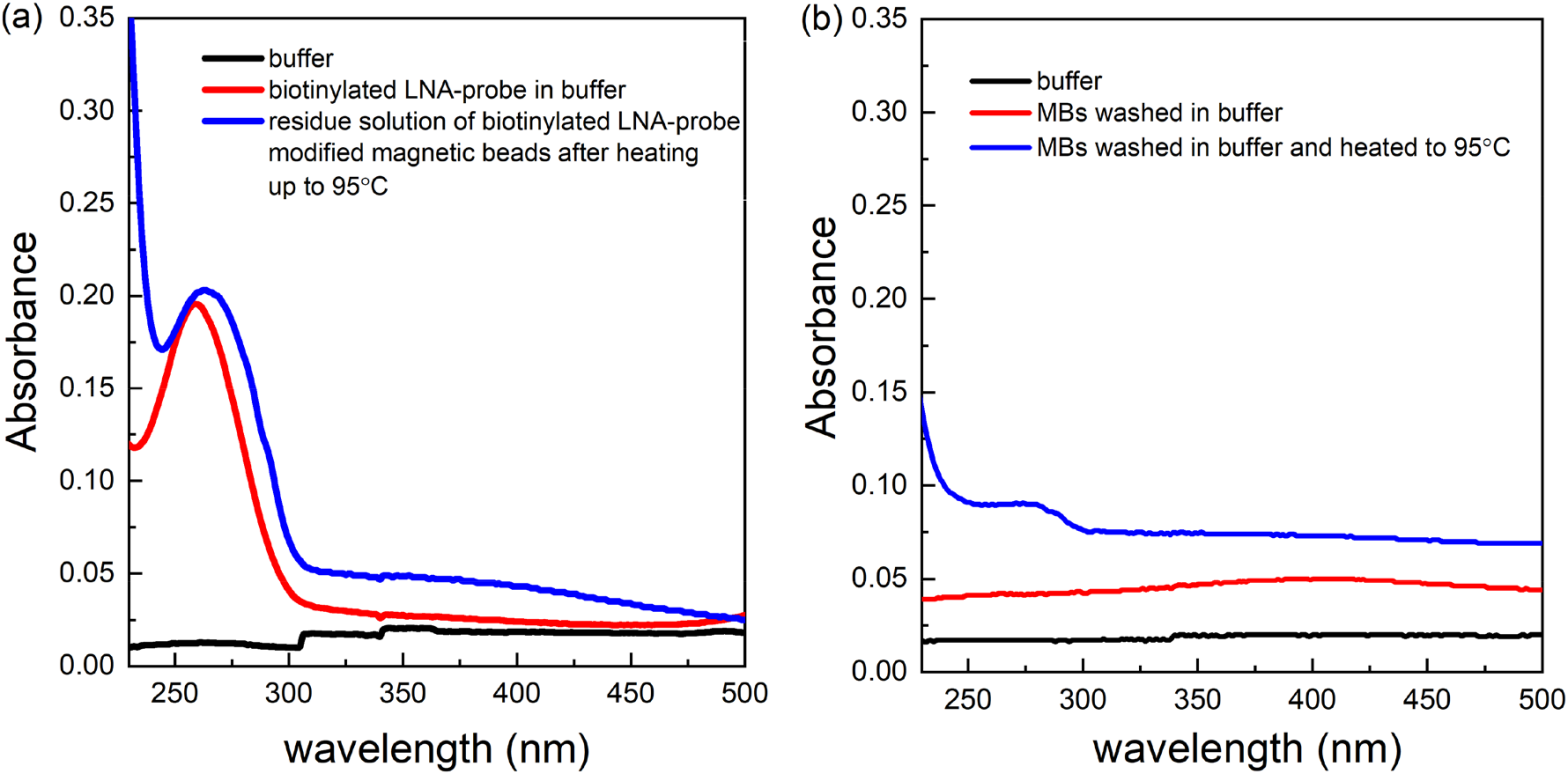
(**a**) A UV absorbance graph for blank buffer (black line), biotinylated LNA probe in buffer (red line), and the residue buffer solution after the heating up of LNA modified magnetic beads at 95 °C for 10 min (blue line). (**b**) UV absorbance graph for blank buffer (black line), residue solution of magnetic beads after being washed in buffer (red line), residue solution of magnetic beads after being washed and heated up at 95 °C for 10 min in buffer (blue line). The absorbance levels below 230 nm were considered as noise due to the increased absorbance of the quarts cuvette itself upon being subjected to light at wavelengths lower than 250 nm.

Following the heating of LNA-modified magnetic beads at 95 °C for 10 min, UV spectrophotometry results in Fig. 3a for the residue solution (blue line) indicated an absorbance peak at 263 nm. This value is very close to the initial absorbance peak, at 259 nm, that was obtained for the LNA probe prior to its immobilization on magnetic beads. Such results could be attributed to the fact that under high temperature conditions, a disruption is made in streptavidin–biotin binding between the magnetic beads and the probe. This results in the discharge of the LNA-probe into solution. Additionally, the slight shift of the absorbance peak (Fig. 3a, red peak → blue peak), after the heating up of the LNA-modified beads, towards the right end of the spectrum could be attributed to the additional discharge of the streptavidin coating of the magnetic beads into solution^40^. This condition was confirmed by a second assay that analyzed the residue solution of magnetic beads (without the LNA probe) before and after heat was applied. The results in Fig. 3b suggest no significant absorbance peak in the residue solution of magnetic beads upon being washed in buffer. This indicates no dissociation of the streptavidin coating from the magnetic beads surface upon washing. However, an absorbance peak at 280 nm was observed within the residue solution after heating up the beads to 95 °C. This could be attributed to the discharge of the streptavidin coating of the magnetic beads upon heating, resulting in the absorbance at 280 nm that indicates the presence of proteins^40^. This also explains the slight shift of the absorbance towards 280 nm (blue peak) in Fig. 3b for the residue solution of LNA-modified magnetic beads upon heating, as the peak corresponds to the dissociation of the LNA-probe together with the streptavidin into solution.

**Figure 4:**
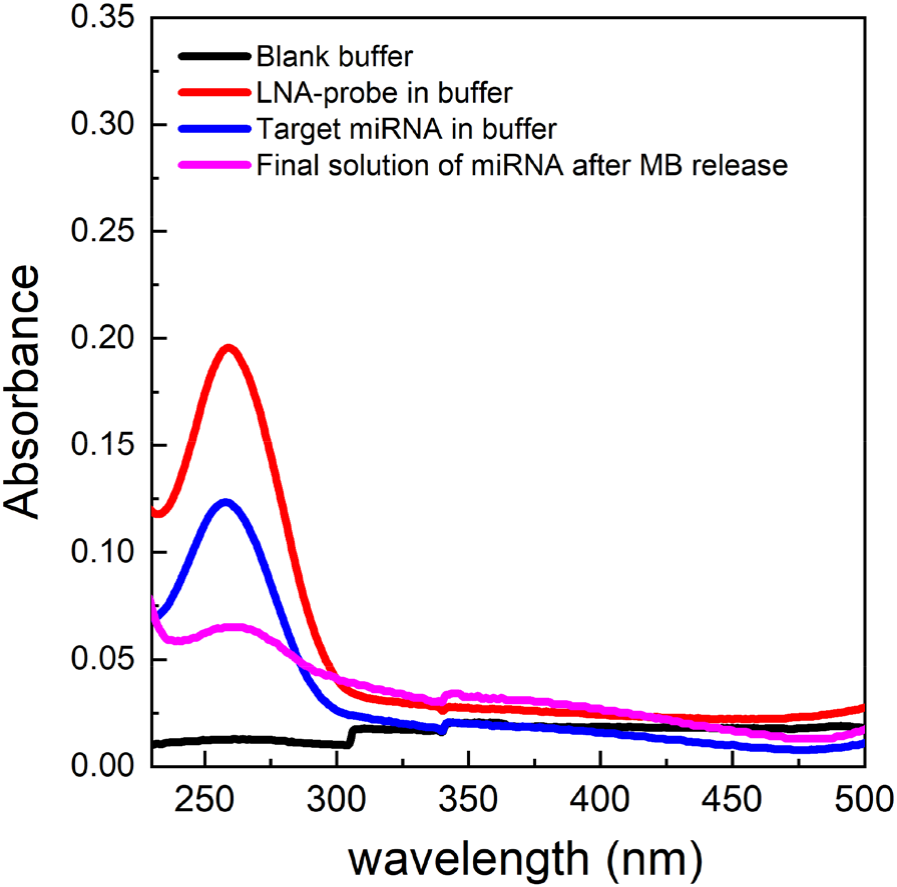
UV absorbance of the biotinylated LNA probe (red), target miRNA (blue), and the miRNA released from the magnetic beads (magenta) using 1 M NaOH. Buffer was set as the baseline measurement (black line) of the spectrophotometer prior to UV analysis. The absorbance levels below 230 nm were considered to be noise due to the increased absorbance of the quarts cuvette itself upon being subjected to light at wavelengths lower than 250 nm.

The results indicated the degradation of the streptavidin coating of magnetic beads upon heating and the crucial need for an alternative method for the denaturing step of the LNA probe-target miRNA duplex that eliminates heat. The efficiency of chemical denaturation was assessed using several methods: incubation with Milli-Q water (ion-free), 50% (w/w) urea solution, 60% DMSO solution and 1 M NaOH (alkaline) solution for 10 min at ambient temperature (see Supplementary Information). The adoption of 1 M NaOH provided the best denaturation of the LNA capture probe-miRNA hybrid duplex with a clear absorbance peak at 260 nm. Therefore, the remaining of the assays were performed by adopting 1 M NaOH as a chemical denaturation method. Figure 4 shows the absorption peaks at 259 nm for the biotinylated LNA probe, at 258 nm for the target miRNA in solution before its capture, and at 259 nm for the solution that contains the captured target miRNA that was released by chemical denaturation in 1 M NaOH solution.

#### Electrochemical impedance spectroscopy (EIS)

Faradaic EIS was selected for the evaluation of the stability of the fabricated PNA-probe prior to analysis with target miRNA. PNA-probes are known for their high specificity to complementary targets and are expected to perform no hybridization with a non-specific target. This was confirmed by an assay that was performed by incubation of PNA-immobilized electrodes with a high concentration (1 nM) of non-specific miRNA target sequences, that resulted in an *R*_ct_ variation of only 0.78 ± 0.07%.

Two assays were then carried out in order to observe the hybridization of the PNA-probe with the target miRNA captured by LNA-modified magnetic beads. The measurements were performed in 10 mM of [Fe(CN)_6_]^3−/4−^ redox couple to monitor the resistance to charge transfer upon target binding events. The first assay adopted the solution of thermally released miRNA from the magnetics beads’ surface for its hybridization on complementary PNA probes. Since high temperatures led to the dissociation of the streptavidin coating from the magnetic beads surface and the release of probe-LNA, no variation of *R*_ct_ was expected due to the lack of a target in the solution. The second assay eliminated the use of high temperatures and adopted a chemical denaturation method (incubation of magnetic beads containing LNA–miRNA helix in 1 M NaOH) for the release of target from the magnetic beads’ surface. Hence, EIS measurements were expected to display an increased value of *R*_ct_ due to the presence of captured miRNA, that is complementary to the probe-PNA. Binding of the target to the PNA-probe is expected to increase the resistance to charge flow (*R*_ct_).

**Figure 5:**
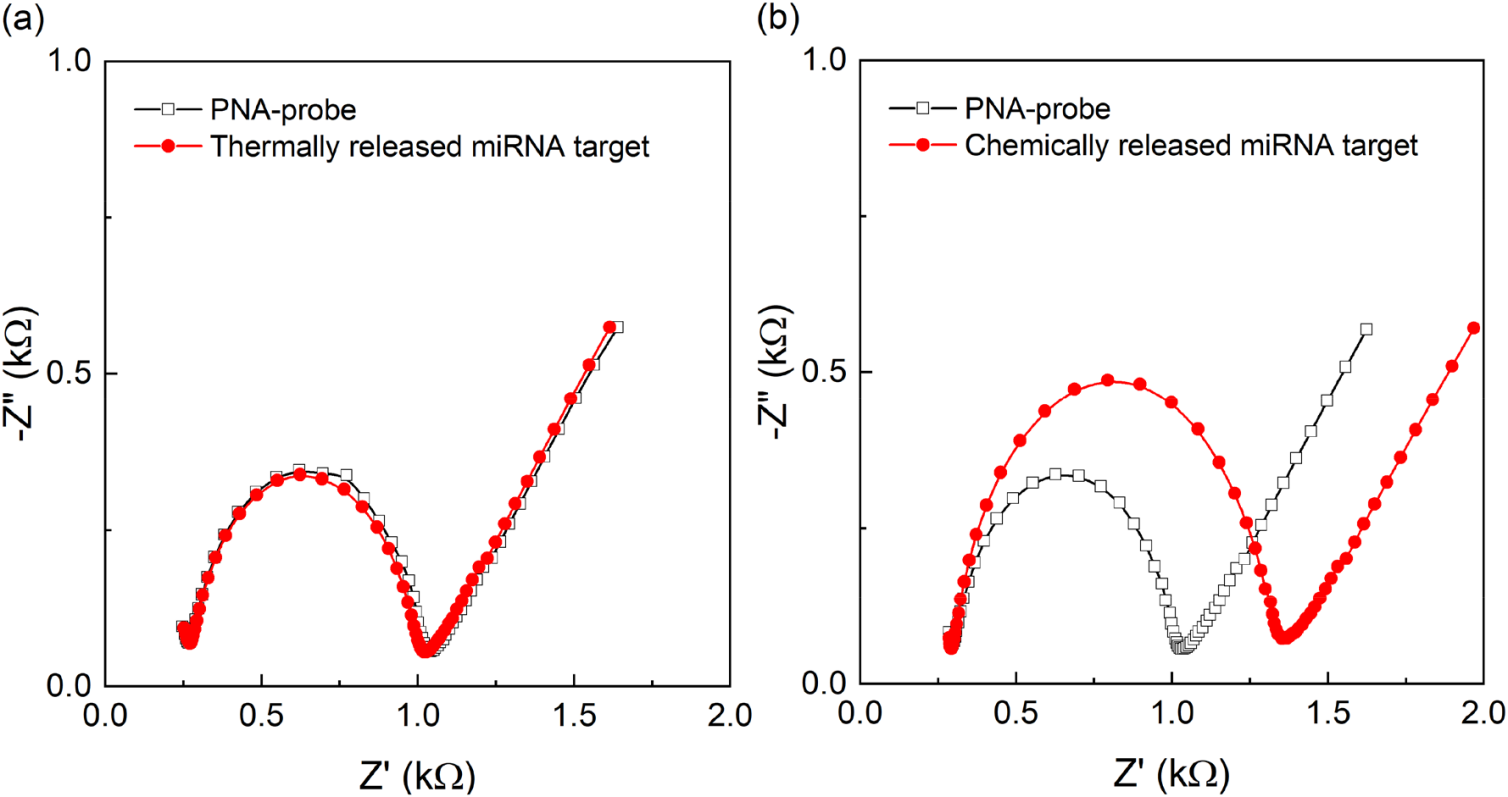
(**a**) EIS characterization of the PNA immobilized probe and its stability in blank buffer followed by hybridization with solution that contains thermally released miRNA from magnetic beads surface. (**b**) EIS characterization of the PNA immobilized probe and its stability in blank buffer followed by hybridization with chemically released miRNA from magnetic beads surface. All measurements were performed in 10 mM PB with 10 mM [Fe(CN)_6_]^3−/4−^.

The results in Fig. 5a represent the measurements of the assay design that used the method of thermal denaturation for the release of target miRNA from the magnetic beads’ surface. A PNA/MCH self-assembled layer with a ratio of 1:10 led to an *R*_ct_ variation of 2.24% upon stabilization, which reflects the formation of a stable PNA-MCH SAM. Initial incubation with the blank buffer showed no significant change in *R*_ct_ (∆*R*_ct_ = 3.8%), which demonstrates a strong stability of the probe before its incubation with the target. This was followed by incubation with the solution that was expected to contain the magnetic beads-captured miRNA, free in solution, upon thermal release. However, the EIS results showed almost no variation in *R*_ct_, (785.6 Ω → 765.9 Ω, ∆*R*_ct_ = 6.2%) upon target incubation, as shown in Fig. 5a. This has once again proven the poor efficiency of thermal release, with possible disruption of the magnetic beads surface, and encourages the application of chemical denaturation methods. As a result, the assay was repeated by replacing thermal denaturation with the incubation in an alkaline solution (1 M NaOH diluted in Milli-Q). The target miRNA that was released into the alkaline solution was in turn re-hybridized with the PNA probe for EIS detection. The conditions for the re-hybridization with the PNA probe was achieved by mixing 10 µl of the target solution (in 1 M NaOH solution) with 100 µl of 10 mM PB solution (at pH 7.3). This is necessary to represent the hybridization buffer and reduce the pH of the alkaline solution for the required hybridization efficiency with the complementary PNA probe. The results shown in Fig. 5b once again suggest the strong stability of PNA-MCH SAM prior to incubation with target. In this case, a significant *R*_ct_ variation of 32.4 ± 5.4% (average over three independent electrodes) was observed after incubation with the target, which corresponds to successful hybridization. This is significantly higher than the blank buffer incubation that had an *R*_ct_ variation of 2.18 ± 0.78%. Hence, our methodology of fishing specific miRNAs from solution by use of LNA-modified magnetic beads was proven to successfully separate the target from solution and its direct electrochemical detection on a PNA-immobilized probe.

## Conclusions

In this work, we developed electrochemical impedance biosensors that can detect the pancreatic cancer biomarker miR-21-5p in diluted serum using PNA-modified biosensors, with a limit of detection of 0.38 fM. In order to further improve the accuracy and sensitivity of the sensors, a new method using magnetic beads was developed to separate and pre-concentrate the target miRNAs from solution. By optimizing the various assay steps, our improved methodology offers direct detection of target miRNAs with no need for pre-labelling of target sequences or enzymatic labelling on the detection probe. It is crucial to develop technologies that provide accurate results without the requirement for many amplification steps. Although the literature offers various magnetic beads-based detection methodologies with great limits of detection, there is still the need for the elimination of a number of the assay steps so that such technologies can be translated into practical point-of-care tests. For this purpose, we have investigated the feasibility of a methodology that eliminates the need for sample preparation, hence the requirement for trained labor, or for additional signal enhancement steps. Additionally, an important and neglected aspect of miRNA detection is distinguishing between the pool of miRNA sequences which can lead to false positives in the results of patients. For this purpose, the LNA-probes that were utilized have a special affinity for complementary miRNAs and offer improved hybridization efficiencies. Such a property of LNA-probes was combined with the separation properties of magnetic beads so that LNA favored capturing of target miRNA from solution was achieved. Following the capture of the target, we have carried out chemical denaturation of the hybridized LNA–miRNA helix for the release of the miRNA from the magnetic beads’ surface. PNA-immobilized gold electrodes were used for the EIS based detection of miRNA, which demonstrates a significant variation in *R*_ct_ (32.4 ± 5.4%) upon target incubation. Although further studies using clinical samples from cancer patients and healthy individuals known to express high or low amounts of miR-21 are required to ensure that the presence of miRNAs competitors does not inhibit the sensitivity and specificity of the method, initial controls with a non-specific miRNA sequence yielded a negligible signal, suggesting the applicability of the methodology. We believe such a model for detection is a crucial step towards the easy fishing of miRNAs from complex solutions, such as blood, and pre-concentration of the target prior to its direct electrochemical detection for quantification.

## Supplementary Information

Supplementary Information.
