# Supplementary material for: Pre-concentration of microRNAs by LNA-modified magnetic beads for enhancement of electrochemical detection
Source: Sci Rep. 2021 Oct 4;11:19650. doi: 10.1038/s41598-021-99145-8 (PMC8490432; doi:10.1038/s41598-021-99145-8)
Supplement: Supplementary file 1 — Supplementary Information. [file 41598_2021_99145_MOESM1_ESM.docx]

**Supplementary Information**

**Pre-concentration of microRNAs by LNA-modified magnetic beads for enhancement of electrochemical detection**

**Serife Ustuner, Mark A. Lindsay, Pedro Estrela**

**1. Optimization of PNA surface density**

Thiol-modified PNA probe and mercaptohexanol were co-immobilized onto gold electrodes and the charge transfer resistance for the electrode surface was determined by electrochemical impedance spectroscopy. For the initial surface chemistry optimization studies, microRNA target concentrations (10 nM and 100 nM) were prepared in 10 mM PB (pH 7.3). The %∆*R*ct increases significantly upon hybridization as the ratio of PNA to MCH functionalized on the surface is reduced gradually from 1:2 to 1:15 in Figure S1. PNA/MCH self-assembled layer with a ratio of 1:15 performed an *R*_ct_ variation of 2.33% ± 2.37% upon stabilization which reflects the formation of a well stable SAM. Hybridization with 10 nM complementary miRNA resulted in 7-fold increase of *R*_ct_, 15.99% ± 2.53%. The best hybridization efficiencies were performed upon adopting 1:10 and 1:15 ratios of PNA to MCH on the surface.

**Figure S1. V**ariation in R_ct_ upon hybridization by adopting various ratios of PNA to MCH on electrode surface.

**2. LNA-miRNA denaturation**

A variety of physical and chemical denaturation methods were investigated for the optimum release of miRNA target from the surface of magnetic beads after being captured by the LNA-probe. This study is crucial for the optimum yield of the target sequence for the detection studies. Thermal and NaOH-based chemical release of miRNAs are described in the main manuscript.

**Removal of Salts**

A low salt concentration can lead to the denaturing of DNA double-strands due to the removal of ions that stabilize the negative charges on each of the two single strands. Electrochemical detection studies have shown that successful hybridization of DNA-DNA [1], DNA-RNA [2], PNA-DNA [3] or LNA-RNA [4] requires the presence of ions that will provide the screening for the negative backbone phosphate groups that are present both in DNA and RNA single strands. Hence, for the denaturation experiments 300 µL of Milli-Q water that was free from salts was added to the tube containing LNA-functionalized magnetic beads previously hybridized with target miRNA. The mixture was incubated at ambient temperature for 10 minutes prior to measurements with UV spectrophotometry.

**Dimethyl Sulfoxide (DMSO)**

DMSO is another agent that has proven to provide efficient denaturation by lowering the melting temperature of the DNA. Upon trying various concentrations of DMSO (25%, 50% and 60%), Wang & Son achieved complete denaturation of DNA in 60% DMSO in one minute followed by further denaturing to 90% over time [5]. On the other hand, another study [6] revealed the denaturation capability of DMSO with higher concentrations and proved that 60% DMSO is sufficient for the complete denaturation of DNA in contrast to low concentrations. Hence, DMSO was purchased from Sigma-Aldrich at a grade for molecular biology use (99.9%). 300 µL of 60% DMSO was prepared in Milli-Q water. The solution was added to the tube containing LNA-functionalized magnetic beads that were previously hybridized with target miRNA. The mixture was incubated at ambient temperature for 10 minutes prior to measurements with UV spectrophotometry.

**Urea**

The denaturation ability of urea has been widely attributed to its capability to disturb interpeptide and interchain hydrogen bonds [7]. Several early studies looking into the effects of urea on suitable model compounds [8] and detergent micelles [9] have suggested this property of urea is grossly oversimplified and at least partly attributed to the destabilization of hydrophobic interactions. Hence, the urea was considered capable of destroying not only hydrogen bonds but also other sources of stabilizations, like hydrophobic interactions. Shen *et al.* adopted a 50% (w/w) urea solution for target DNA dehybridization on the surface of magnetic submicrobeads following their capture with DNA-probes [10]. The technique achieved a limit of detection of 8.5 fM. In addition, Zhang *et al.* also used a 50% (w/w) urea solution to release target DNA from the surface of magnetic nanobeads immobilized with probe DNA [11]. Hence, we employed the same methodology and 300 µL of 50% (w/w) urea were prepared for incubation in a tube containing LNA-functionalized magnetic beads previously hybridized with target miRNA. The mixture was incubated at ambient temperature for 10 minutes prior to measurements with UV spectrophotometry.

**3. The use of UV-Spectrophotometry for validation of design steps**

The results indicated the degradation of the streptavidin coating of magnetic beads upon heating and the crucial need for an alternative method for the denaturing step of the LNA probe-target miRNA duplex that eliminates heat. Therefore the efficiency of denaturation was assessed using several chemical methods: incubation with Milli-Q water (ion-free), 50% (w/w) urea solution, 60% DMSO solution and 1 M NaOH (alkaline) solution for 10 min at ambient temperature.

The results in Figure S2 indicated the degradation of the streptavidin coating of magnetic beads upon heating and the crucial need for an alternative method for the denaturing step of the LNA probe-target miRNA duplex that eliminates heat. For this purpose, another set of assays were performed which compared the efficiency of denaturation and target release using a number of chemical methods. Four different methods were adopted: incubation with Milli-Q water (ion-free), 50% (w/w) urea solution, 60% DMSO solution and 1 M NaOH (alkaline) solution for 10 minutes at ambient temperature Prior to the assays, the LNA-functionalized magnetic beads were incubated with each solution in order to confirm that the biotin-streptavidin bonding between the probe and the magnetic beads was not disrupted upon incubation with these solutions. Hence, the absorbance peaks observed in the residue solution after denaturation of the LNA probe functionalized magnetic beads-target miRNA can be attributed only to target release and no interference due to probe dissociation from the magnetic beads surface into solution.


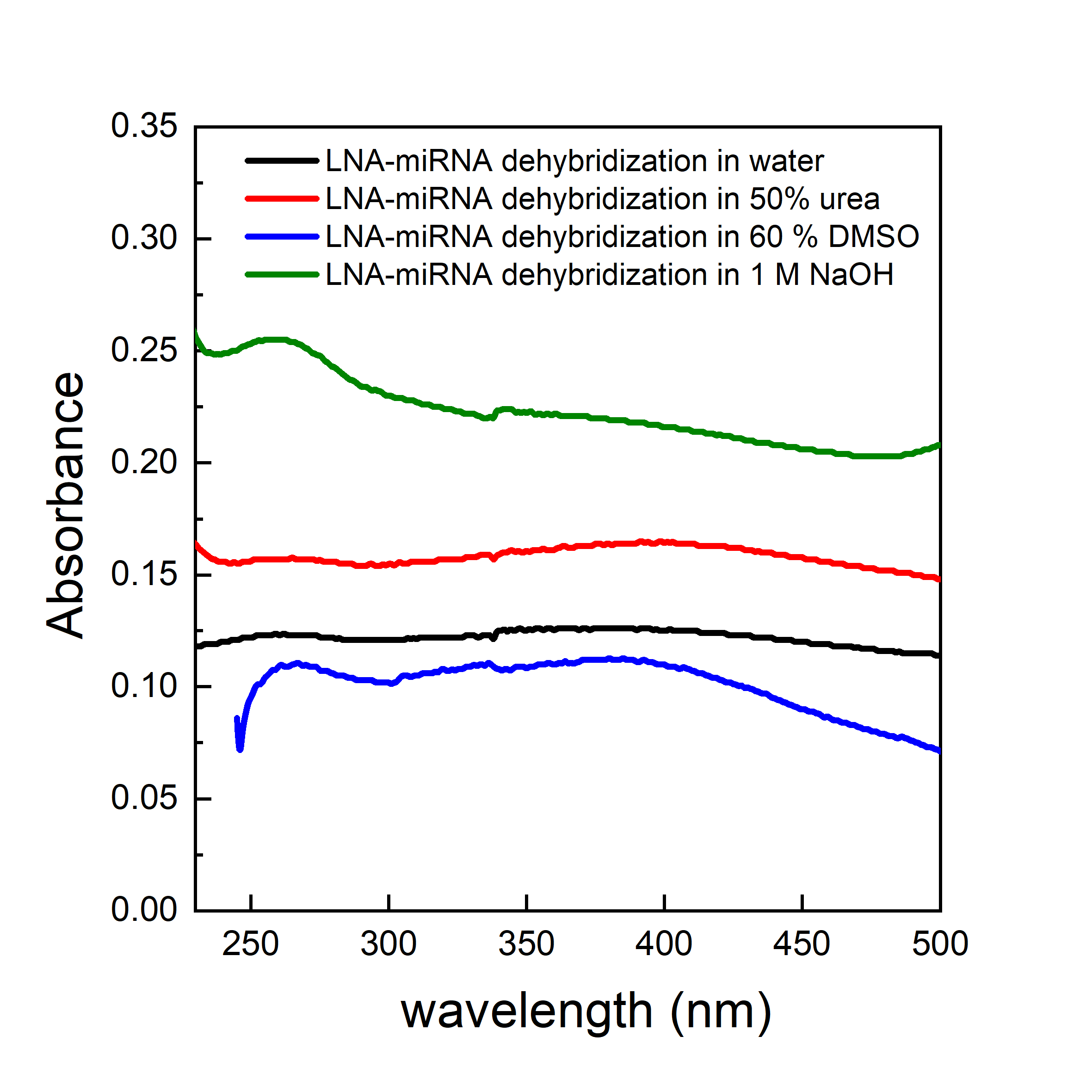


**Figure S2.** UV-absorbance graph of the final solution of target miRNA after performing dehybridization in Milli-Q water (black line), in 50% (w/w) urea solution (red line), in 60% DMSO solution (blue line), and in 1 M NaOH solution (green line) for 10 min. For each measurement, a baseline measurement was carried out within the solution of investigation prior to the UV-analysis. The absorbance levels below 230 nm were considered to be noise due to the increased absorbance of the quarts cuvette itself upon being subjected to light at wavelengths lower than 250 nm.

As suggested by the results in Figure S2, a distinguishable nucleic acid absorbance peak was observed only in two cases: one with the adoption of 1 M NaOH, and one with the adoption of 60% DMSO for the denaturation of the LNA capture probe-miRNA hybrid duplex. The peak is most significant and clear at 260 nm for the case of 1 M NaOH where this peak is slightly shifted to 264 nm upon the adoption of 60% DMSO which also presented very noisy data upon being measured.

**References**

[1] Keighley, S.D., Li, P., Estrela, P. & Migliorato, P. Optimization of DNA immobilization on gold electrodes for label-free detection by electrochemical impedance spectroscopy. *Biosens. Bioelectron.* **23**, 1291–1297; 10.1016/j.bios.2007.11.012 (2008).

[2] Wang, J., Kawde, A.N., Erdem, A. & Salazar, M. Magnetic bead-based label-free electrochemical detection of DNA hybridization. *Analyst* **126**, 2020–2024; 10.1039/b106343j (2001).

[3] Keighley, S.D., Estrela P., Li. P. & Migliorato P. Optimization of label-free DNA detection with electrochemical impedance spectroscopy using PNA probes. *Biosens. Bioelectron.* **24**, 906–911; 10.1016/j.bios.2008.07.041 (2008).

[4] Laschi, S., Palchetti, I., Marrazza, G. & Mascini, M. Enzyme-amplified electrochemical hybridization assay based on PNA, LNA and DNA probe-modified micro-magnetic beads. *Bioelectrochemistry* **76**, 214–220; 10.1016/j.bioelechem.2009.02.012 (2009).

[5] Wang, X. & Son, A. Effects of pretreatment on the denaturation and fragmentation of genomic DNA for DNA hybridization. *Environ. Sci. Processes Impacts* **15**, 2204–2212; 10.1039/c3em00457k (2013).

[6] Wang, X., Lim, H.J. & Son, A. Characterization of denaturation and renaturation of DNA for DNA hybridization. *Environ. Health Toxicol.* **29**, 1–8; 10.5620/eht.2014.29.e2014007 (2014).

[7] Herskovits, T.T. Nonaqueous solutions of DNA; denaturation by urea and its methyl derivatives. *Biochemistry* **2**, 335–340; 10.1021/bi00902a027 (1963).

[8] Levy, M., & Magoulas, J.P. Effect of urea on hydrogen bonding in some dicarboxylic acids. *J. Am. Chem. Soc.* **84**, 1345–1349; 10.1021/ja00867a003 (1962).

[9] Bruning, W., & Holtzer, A. The effect of urea on hydrophobic bonds: the critical micelle concentration of n-dodecyltrimethylammonium bromide in aqueous solutions of urea. *J. Am. Chem. Soc.* **83**, 4865–4866; 10.1021/ja01484a044 (1961).

[10] Shen, L., Zhang, X., & Jin, W. Signal amplification based on DNA hybridization – dehybridization reaction on the surface of magnet submicrobeads for ultrasensitive DNA detection. *Analyst* **137**, 4849–4854; 10.1039/c2an35587f (2012).

[11] Zhang, X. Li, L., Li, L., Chen, J., Zhou, G., Si, Z., & Jin, W. Ultrasensitive electrochemical DNA assay based on counting of single magnetic nanobeads by a combination of DNA amplification and enzyme amplification. *Anal. Chem.* **81**, 8083–8089; 10.1021/ac802183u (2009).
